# The strategy and clinical relevance of in vitro models of MAP resistance in osteosarcoma: a systematic review

**DOI:** 10.1038/s41388-022-02529-x

**Published:** 2022-11-25

**Authors:** Victoria L. Tippett, Luke Tattersall, Norain B. Ab Latif, Karan M. Shah, Michelle A. Lawson, Alison Gartland

**Affiliations:** 1 The Mellanby Centre for Musculoskeletal Research, Department of Oncology and Metabolism, The University of Sheffield, Beech Hill Road, Sheffield, S10 2RX UK; 2 Universiti Kuala Lumpur Royal College of Medicine Perak, No. 3 Jalan Greentown, 30450 Ipoh, Perak Malaysia

**Keywords:** Bone cancer, Cancer models

## Abstract

Over the last 40 years osteosarcoma (OS) survival has stagnated with patients commonly resistant to neoadjuvant MAP chemotherapy involving high dose methotrexate, adriamycin (doxorubicin) and platinum (cisplatin). Due to the rarity of OS, the generation of relevant cell models as tools for drug discovery is paramount to tackling this issue. Four literature databases were systematically searched using pre-determined search terms to identify MAP resistant OS cell lines and patients. Drug exposure strategies used to develop cell models of resistance and the impact of these on the differential expression of resistance associated genes, proteins and non-coding RNAs are reported. A comparison to clinical studies in relation to chemotherapy response, relapse and metastasis was then made. The search retrieved 1891 papers of which 52 were relevant. Commonly, cell lines were derived from Caucasian patients with epithelial or fibroblastic subtypes. The strategy for model development varied with most opting for continuous over pulsed chemotherapy exposure. A diverse resistance level was observed between models (2.2–338 fold) with 63% of models exceeding clinically reported resistance levels which may affect the expression of chemoresistance factors. In vitro p-glycoprotein overexpression is a key resistance mechanism; however, from the available literature to date this does not translate to innate resistance in patients. The selection of models with a lower fold resistance may better reflect the clinical situation. A comparison of standardised strategies in models and variants should be performed to determine their impact on resistance markers. Clinical studies are required to determine the impact of resistance markers identified in vitro in poor responders to MAP treatment, specifically with respect to innate and acquired resistance. A shift from seeking disputed and undruggable mechanisms to clinically relevant resistance mechanisms may identify key resistance markers that can be targeted for patient benefit after a 40-year wait.

## Introduction

Osteosarcoma (OS) is the most common malignant bone cancer and affects approximately 2.5 per million people in England equivalent to 135 cases per year [1]. OS is more common in males [1–3] and a higher incidence rate has been observed in Black patients [2, 3]. The peak age at diagnosis of bone sarcoma for females and males is 13 and 15–17 years, respectively [2]. This reflects a pattern of disease progression in line with growth, with bone development occurring approximately 2 years earlier in pubescent females than males [4]. A second peak is also evident in those above 65 years and is associated with Paget’s disease [1, 3, 5], secondary cancer [3] and a poorer outcome [5].

The 5-year survival rate increased from 17% [6] to 68% [7] for OS patients with localised disease during the 1970s when chemotherapy was introduced into practice. However, whilst the average 5-year survival for all cancer patients increased by around 20% from 1980 to 2010 [8], 5-year overall survival and recurrence rates for localised OS have stagnated since the 1980s [9]. This is despite an increase in the rate of limb-salvage surgery owing to advancement in surgical technique and earlier detection [9]. This lack of progress is impacted by an absence of improved treatment options over the last 40 years with approximately one third of patients relapsing commonly more than once [10, 11], with overall survival for these patients reported at 23–29% [10, 12]. In addition, 16% of patients have detectable metastases at diagnosis and up to 77% of these will succumb to the disease within 5 years [13].

Although the introduction of chemotherapy drastically changed the extremely low survival rates achieved with surgery alone, OS is regarded as relatively chemoresistant as many single agents have shown poor responses in patients. The drugs methotrexate (at a high dose, hdMTX), doxorubicin (DOX), cisplatin (CDDP) and ifosfamide (IFOS), collectively known as MAPi, have the highest single agent response rates ranging from 30 to 40% [14]. Current therapy therefore involves a combination of these agents, with patients receiving two 5-week neoadjuvant cycles followed by a further 4–6 adjuvant cycles as part of the widely adopted EURAMOS-1 protocol [15]. Multiagent chemotherapy is used to circumvent a single resistance mechanism as each agent has a unique target. Specifically, the alkylating agent CDDP enters cells via passive diffusion [16] and creates inter- and intra-strand DNA adducts that induce apoptosis [17]. The alkylating agent IFOS which is a derivative of nitrogen mustard also induces DNA damage similarly through cross-linking [18]. The anthracycline DOX isolated from Streptomyces peucetius var. caesius [19] enters cells through passive diffusion and active transport [20]. Several mechanisms of action have been proposed for DOX including; free radical generation, inhibition of topoisomerase II-mediated DNA repair and DNA intercalation and this may be concentration dependent [21]. MTX can be transported into cells via various transporters of the solute carrier family, the most recognised of which is reduced folate carrier (*SLC19A1*/RFC1). Once inside the cell this antimetabolite is converted into a more active form where it inhibits dihydrofolate reductase (DHFR) by mimicking folic acid and suppresses thymidine and purine synthesis, preventing proliferation and leading to cell death [22].

In order to clinically measure the effectiveness of a chemotherapy regimen, samples of the tumour surgically removed after neoadjuvant therapy are used to determine percentage necrosis. An established threshold for necrosis is ‘good responder’ >90% and ‘poor responder’ <90% based on its prediction of patient outcome. In 22 OS patients treated with MAPi, percentage necrosis was found to predict 5-year metastasis and recurrence-free survival by univariate analysis [23]. Similarly, in 38 patients necrosis was the only factor at bivariate analysis capable of predicting survival and recurrence [24]. In a larger cohort of 881 OS patients necrosis was again the only factor able to predict patient outcome by multivariate analysis and was linked to shorter; 5-year disease-free survival, overall survival, rate of limb-salvage surgery and time to relapse or death, in addition to an increased rate of distant relapse [25]. Percentage necrosis is therefore a key indicator of chemotherapy response and is associated with several crucial patient outcome measures including survival, relapse and metastasis at multiple levels of analysis.

The major challenge in OS treatment is poor responders, with the exact mechanisms of how a patient develops chemoresistance being unknown. This resistance can be defined as acquired or intrinsic, with these terms denoting tumours that become unresponsive after initially responding and those that fail to respond to initial treatment, respectively. Acquired resistance suggests the development of genetic alterations in response to chemotherapy resulting in the formation of a subset of resistant cells, whilst intrinsic resistance indicates a pre-existing survival benefit to subpopulations of cells due to inherent heterogeneity. Resistance to the designated MAP regimen is common with 34–68% [10, 26] of OS patients classed as poor responders to neoadjuvant therapy, potentially indicating high intrinsic resistance in OS. These patients have a greater risk of relapse and rely on aggressive second-line therapy and repeated surgical intervention for survival [10, 12]. However, there is no consensus for second-line therapy in OS, with studies that modify adjuvant treatment by dose intensification [27] or the addition of agents with diverse targets [25] failing to demonstrate a survival benefit in poor responders, providing extra weight to the claim of broad innate resistance in these patients. One study also identified that despite an initial sensitivity to DOX in 82% of biopsy samples, 46% of these patients acquired resistance during MAPi treatment and were termed poor responders [28]. Together this demonstrates the importance of both innate and acquired resistance in OS and highlights the pressing demand for researchers to uncover mechanisms of MAP resistance that can be targeted to treat the large group of patients overlooked by current treatment standards.

In order to effectively research mechanisms of chemoresistance and due to the rarity of OS in the population, relevant in vitro models are required. Different initial drug concentrations have been suggested to recreate intrinsic and acquired chemoresistance in vitro. For example, resistance can be acquired by exposure to low doses for prolonged periods of time; whilst high initial concentrations may select for an intrinsically resistant subpopulation [29]. Further considerations include creating either a clinically relevant or a high-level laboratory model. Clinically relevant models attempt to mimic the dose, fold resistance and exposure method achieved clinically and are theorised to develop resistance mechanisms that more closely resemble those found in patients. The peak plasma concentration has been suggested as a guide to achieve a clinically relevant dose in vitro [29, 30]. Clinically relevant levels of resistance are reportedly low and range from 2- to 5-fold amongst lung, ovarian and neuroblastoma patients [30]. This has also been mirrored in a small number of acquired and intrinsically resistant primary sarcoma cell lines that display up to 6.1-fold resistance to DOX compared to normal fibroblasts [31]. As OS patients treated with the EURAMOS-1 protocol are exposed to the MAP regime over a short period of 4–72 h accompanied by a prolonged interval between treatments [15], a pulsing strategy is suggested to mimic this more closely [29, 30]. Generally, the pulse method uses exposure to high concentrations followed by a drug-free interval. In contrast, laboratory models aim to generate a high level of resistance that is stable upon drug withdrawal and this is achieved by continuous exposure to low concentrations followed by stepwise dose escalation [29, 30].

Different exposure strategies have been reported to generate unique resistance mechanisms within the same cell line. For example in a chemoresistant ovarian model generated by a 2 h pulse in 100 µM CDDP downregulation of the transporters ATP binding cassette subfamily C member 1 (*ABCC1*/MRP1) and lung resistance related protein 1 (*MVP*/LRP1) was observed, however this was not found in a second model exposed to 10–80 µM CDDP over a period of 48 h [32]. In addition, in acute lymphoblastic leukaemia cells continuously exposed to MTX from 5 nM to 50 µM, upregulation of *DHFR* and downregulation of *SLC19A1*/RFC1 conferred resistance via decreased accumulation and target overexpression. In contrast, those produced via 24 h pulsing in 10–50 µM had lower levels of polyglutamates that induced resistance by reducing MTX retention and activity [33]. Together this demonstrates that multiple exposure strategies can be used to generate chemoresistant models with variation in the resulting chemoresistant mechanisms produced. A consideration for the differences in these strategies should be taken in order to identify clinically relevant resistance markers that can be targeted to urgently tackle the issue of chemoresistance faced by so many OS patients.

This comprehensive systematic review scoped the literature to summarise methods used to generate chemoresistant OS cell lines and the potential effects this may have on the expression of resistance associated genes, proteins and non-coding RNA (ncRNA). A comparison of intrinsic resistance markers to those found in OS patients with a poor response to chemotherapy, greater relapse or metastasis allowed the clinical relevance of current in vitro models to be determined whilst highlighting strategies to improve the clinical relevance of future models.

## Results

### Overall study quality and bias

A total of 52 studies were entered into this review with 36 assessing differentially expressed markers using in vitro models of OS MAP resistance and 18 investigating expression in relation to defined outcomes in OS patients treated with MAP therapy with a cross-over of two studies that addressed both. Overall, six in vitro studies were rated high quality with the remaining 30 scored as moderate (Table 1). Of note in vitro studies universally scored low quality for their methodology due to an incomplete description of model development. All in vitro studies showed no indication of selection bias, however bias was recognised in the categories for detection, attrition and reporting. In comparison, one and 17 clinical studies were regarded as high and moderate quality, respectively. Most clinical studies demonstrated a higher risk of bias in selection criteria with baseline characteristics of patient outcome groups often incompletely described.

**Table 1:**
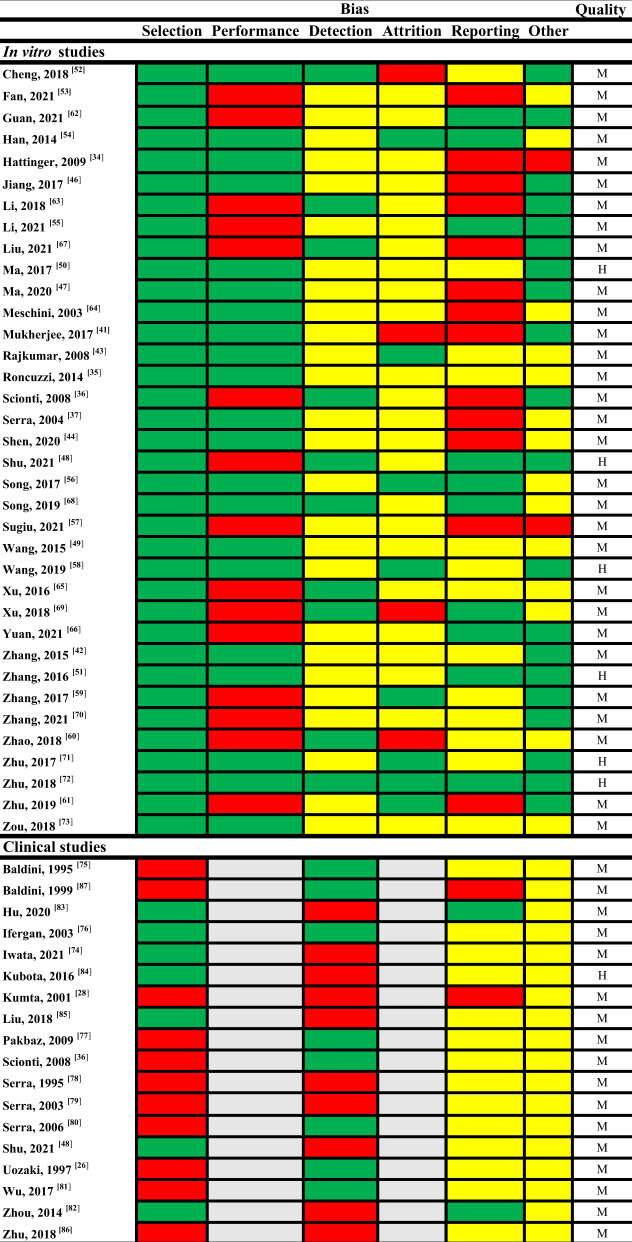
Risk of bias and quality assessment.

In vitro (36) and clinical (18) studies were assessed for risk of bias and quality. For bias: Green, low. Yellow, medium. Red, high. Grey, NA. For quality: M = moderate (4–7), H = high (≥8).

### Cell model source

Included in this review are 36 in vitro studies with a publication date ranging from 2003 to 2021. As indicated in Table 2 it is common for studies to use more than one cell line and to share use of the same models with a total of 41 distinct chemoresistant models entered into this review. Seven models were used multiple times between studies allowing further verification or identification of more diverse resistance mechanisms. Four studies investigated variants with increasing levels of resistance that were produced during model development [34–37]. The fibroblastic MG63 cell line obtained from a 14-year-old Caucasian male [38] was the most commonly reported in a total of 17 studies encompassing 11 DOX resistant, one MTX resistant and four CDDP resistant models with a single CDDP resistant model shared amongst three studies. The epithelial U2OS cell line was used as a model in 15 studies and was derived from the tibia of a 15-year-old Caucasian female patient during amputation [39]. The epithelial SAOS2 cell line established from an 11-year-old Caucasian female treated with combination therapy including MTX and DOX [40] was used in 11 studies. The KHOS cell line, derived from the female HOS line after transformation with the Kirsten murine sarcoma virus, was used in eight studies found via this review with six sharing the same DOX resistant model making it the most widely researched model within the field. The HOS, 143b and SOSP-9607 cell lines were used in three, one and one studies, respectively.

**Table 2:** Methodology for the in vitro development of MAP resistant models in OS.

	Dose response exposure time (h)	EC_50_ (µM)	Cell line	Model exposure method (–h)	Starting conc. (µM)	Final conc. (µM)	RI	Variants	Reference	Model
MTX	24	25.78	SAOS2	P-24	1.1	4.4	12.7	NS	[49]	Wang_SAOS2/MTX-P
	48	0.35	MG63	P-48	0.22	22	112.4	NS	[69]	Xu_MG63/MTX-P
		0.47	U2OS				93.1			Xu_U2OS/MTX-P
	96	0.03	SAOS2	C	0.0066	0.066–2.2	15–281	Y	[34, 36, 37]	Serra_SAOS2/MTX-C
		0.01	U2OS			0.0066–2.2	3^c^–135			Serra_U2OS/MTX-C
DOX	3	0.4	143b	P-3	NS	100	187.5	NS	[43]	Rajkumar_143B/DOX-P
	NS	0.14	U2OS	P-24	0.345	NS	95.6	NS	[44]	Shen_U2OS/DOX-P
		0.18	MG63				72.9			Shen_MG63/DOX-P
	48	1.8	HOS	C	0.01	1	11.28	NS	[52]	Cheng_HOS/DOX-C
		2.1	MG63				12.8			Cheng_MG63/DOX-C
	96	0.013	SAOS2	C	0.0052	0.0517–1	72–338	Y	[34, 64, 97]	Serra_SAOS2/DOX-C
		0.01	U2OS				15–330			Serra_U2OS/DOX-C
	48	1.6	KHOS	C	0.0035	0.035	6.3	NS	[50, 51, 59, 61, 71, 72, 120]	Lourda_KHOS/DOX-C
		10.78	U2OS				15			Lourda_U2OS/DOX-C
	48	11.15	MG63	C	1	10	4.5^c^	NS	[47]	Ma_MG63/DOX-C
	72	0.035	MG63	C	0.0517	0.0517–0.1724	10–28	Y	[35]	Roncuzzi_MG63/DOX-C
	48	1.1	MG63	C	0.0043	1.724	10.5	NS	[58]	Wang_MG63/DOX-C
	NS	5.52	MG63	C	0.01	0.1	6^c^	NS	[65]	Xu_MG63/DOX-C
	72	0.006	MG63	C	0.005	0.065	18.2	NS	[42]	Zhang_MG63/DOX-C
	24^a^	1.15	U2OS	C	0.015	0.12	3^c^	NS	[48]	Shu_U2OS/DOX-C
		0.93	HOS				3.5^c^			Shu_HOS/DOX-C
	NS	2.01	KHOS	C	NS	NS	5.6^c^	NS	[62]	Guan_KHOS/DOX-C
		4.33	MG63				6.9			Guan_MG63/DOX-C
	24	0.03	U2OS	C	NS	NS	30.7	NS	[57]	Sugiu_U2OS/DOX-C
		0.025	MG63				19.2			Sugiu_MG63/DOX-C
	48	0.226	SAOS2	C	0.001	5	46.8	NS	[67]	Liu_SAOS2/DOX-C
	48	3.5	MG63	C	NS	NS	8	NS	[66]	Yuan_MG63/DOX-C
		3.5	KHOS				3.3^c^			Yuan_KHOS/DOX-C
	24	2.87	MG63	C	0.005	0.1	7.5	NS	[55]	Xia_MG63/DOX-C
CDDP	NS	1.8	SAOS2	P-72	1	50	4.44^c^	NS	[53]	Fan_SAOS2/CDDP-P
	72	8.17	SOSP-9607	P-48	0.333	6.66	6.3	NS	[54]	Han_SOSP9607/CDDP-P
	24	35	HOS	P-2	3333	3333	2.2^c^	NS	[41]	Mukherjee_HOS/CDDP-P
	24^b^	9	SAOS2	P-24	1.5	16	3.4^c^	NS	[56, 68, 70]	Song_SAOS2/CDDP-P
		10	MG63				3.2^c^			Song_MG63/CDDP-P
	NS	5	MG63	P-72	0.1	0.2	4^c^	NS	[73]	Zou_MG63/CDDP-P
	72	9.04	U2OS	C	10	10	14.1	NS	[46]	Jiang_U2OS/CDDP-C
		8.35	MG63				14.5			Jiang_MG63/CDDP-C
	72^b^	3.33	SAOS2	C	0.333	3.33	3^c^	NS	[63]	Li_SAOS2/CDDP-C
	48	0.85	MG63	C	0.5	2	6.1^c^	NS	[47]	Ma_MG63/CDDP-C
	NS	NS	SAOS	C	1	3.33	4^c^	NS	[60]	Zhao_SAOS2/CDDP-C
			U2OS				5^c^			Zhao_U2OS/CDDP-C

Data extracted from in vitro studies included in this review. The concentration that elicits a half maximal response (EC_50_) is stated for the parental cell line. Resistance index (RI) indicates the fold resistance to chemotherapy.

*P* pulse, *C* continuous, *NS* not stated in paper.

^a^Pulsing strategy was used for the dose response experiment where cells were exposed for only 24 h of the total 72 h assay.

^b^Multiple timepoints used for dose response experiments.

^c^Clinically relevant levels of resistance.

### Pulsed vs continuous exposure for model development

For the purpose of this review, we defined a pulsed strategy as a fixed exposure period followed by a defined drug-free recovery. Continuous models were those described as continuously treated without a defined exposure period. Out of the 41 distinct models listed in Table 2, 12 were generated by pulsed exposure ranging from 2 to 72 h and the remaining 29 models were established by continuous treatment. The majority of the 24 DOX resistant models were treated continuously (87.5%), whereas the two strategies were used equally amongst the 12 CDDP-resistant models, with a pulse strategy dominating for the five MTX resistant models. Three models used a constant drug concentration whilst the remaining 38 models were generated by stepwise dose escalation including nine models where the initial or final concentrations were not reported. One study described pulsing in a clinically relevant concentration of CDDP, however the determination of this was not provided [41]. For the purpose of this review a resistance index (RI) of ≤6.1 identified in sarcoma patients previously [31] was used as a maximum threshold for a clinically relevant level of resistance with all MTX models,18/24 DOX models and 3/12 CDDP models exceeding this.

### Drug concentrations, EC_50_ and fold resistance

For model generation, most studies used a starting concentration less than the EC_50_ of the parental cell line; however, the EC_50_ varied greatly due to differences in the exposure time used for determination. For example, one study used a 96 h exposure, four used 72 h, seven used 48 h, four used 24 h, one used 3 h and one study used a 24 h pulse followed by two days of drug-free culture. Two additional studies determined the EC_50_ at multiple timepoints with the remaining 16 studies not clearly stating the exposure period. For all models the EC_50_ was determined from either colorimetric or dye exclusion viability assays.

For MTX the EC_50_ in parental cell lines ranged from 25.78 µM after 24 h, to 0.35–0.47 µM after 48 h and to 0.01–0.03 µM after 96 h. The starting concentration ranged from 0.0066 to 1.1 µM and the final concentration from 2.2 to 22 µM, respectively, with a fold increase of 4–333. These concentrations were higher for pulse models (0.22–1.1 to 4.4–22 µM) compared to continuous models (0.0066 to 0.066–2.2 µM). Variants were produced in the two distinct continuous models ‘Serra_U2OS/MTX-C’ and ‘Serra_SAOS2/MTX-C’ with the earliest variants displaying an RI of three and 15, respectively.

For DOX the EC_50_ was 0.4 µM after 3 h, 2.87 µM after 24 h, 0.226–11.15 µM after 48 h and 0.0055–0.0345 µM after 72 h. Amongst studies that used the same cell line and exposure period the EC_50_ differed, for example in the MG63 cell line DOX exposure for 72 h gave an EC_50_ ranging from 0.0055 to 0.0345 µM [35, 42]. This may be explained by the different methods used to analyse viability with one study using the formation of formazan as a colorimetric measure of metabolic activity and the other using erythrosine B dye exclusion as a measure of cell membrane damage. The starting concentration for continuous models ranged from 0.001 to 1 µM and this increased to a final concentration of 0.035 to 10 µM, with a fold increase of 3–5000. Due to a lack of standardised reporting, the two studies that included pulsed models failed to state both their initial and final concentrations [43, 44]. Variants were created for the three continuous models ‘Serra_U2OS/DOX-C’, ‘Serra_SAOS2/DOX-C’ and ‘Roncuzzi_MG63/DOX-C’ with the earliest variants of each model displaying an RI of 15, 72 and 10, respectively.

For CDDP the EC_50_ was 9–35 µM after 24 h, 0.85 µM after 48 h and to 3–9 µM after 72 h. The starting concentration for continuous models ranged from 0.333 to 10 µM and this was increased to a final concentration of 2–10 µM, a difference of 1–10 fold. Pulsed models started at 0.1–1.5 µM and this was increased to 0.2–50 µM, a difference of 1-50 fold. However, one study determined an EC_50_ of 35 µM after 24 h in the HOS cell line and generated low level resistance by pulsing in 3.33 mM for 2 h. In this study CDDP was dissolved in DMSO [41], which reduces cytotoxicity due to the displacement of chloride ligands by water [45] suggesting this may have contributed to the greater EC_50_ recorded and the comparatively inflated concentration used. One study reported an EC_50_ of 8.35 µM after 72 h in MG63 cells [46] whilst another recorded an EC_50_ of 0.85 µM after 48 h [47]. The reason for this discrepancy is unclear as both studies used colorimetric endpoints and neither stated their choice of diluent.

The resistance level varied greatly from 3 to 338 fold for DOX [34, 48], 2.2 to 14.5 fold for CDDP [41, 46] and 12.7 to 281 fold for MTX [37, 49]. Rarely studies chose to analyse variants of their model produced at earlier developmental stages to identify differences in expression as resistance increases. Fifteen models plus one early variant demonstrated a clinically relevant level of resistance at ≤6.1 and these were distributed across 11 continuous and five pulsed models. The majority (63%) of models therefore exceeded a clinically relevant level of resistance. The stability of resistance was explored only in a single study where cells were cultured drug-free for 1 month although no data was shown to verify this [48]. Eighteen studies stated culture conditions used for the maintenance of their model. This involved exposure to a lower dose than the final concentration used for development for all pulsed models. For continuous models a maintenance concentration that equalled or exceeded the final concentration was often used.

### Multi-drug resistance

In 13 distinct models across nine in vitro studies cross-resistance to eight chemotherapy agents MTX, CDDP, DOX, trimetrexate (TRIM), epirubicin (EPI), theprubicin (THP), paclitaxel (PAC) and 5-fluorouracil (5-FU) was investigated (Table 3). Of these models, five were resistant to two MAP agents; however, four of these did not assess cross-resistance to all MAP agents. As MAP therapy is the most widely used to treat OS patients this identifies crucial missing information regarding model characterisation. In contrast, six models were fully characterised and found to be resistant to all MAP agents. In these models the RI was generally low ranging from 6 to 15 apart from a single model generated by MTX treatment that recorded an RI of 281. DOX resistant U2OS and SAOS2 variants with low resistance (RI ≤ 15) remain susceptible to MTX compared to later variants with elevated resistance [34]. Likewise, in an MTX resistant SAOS2 model cross-resistance to CDDP, DOX and TRIM was only evident in variants with high resistance (RI ≥ 24) [37]. In contrast, cross-resistance to DOX failed to develop for all MTX resistant U2OS variants (RI = 3–135) [34].

**Table 3:** Multidrug resistance in in vitro models.

Model	Treatment	RI	Drugs tested for cross-resistance		*ABCB1*/PGP overexpression	Reference
			No cross-	Cross-resistance		
			resistance			
**Serra_U2OS/MTX-C**	MTX	135	DOX	TRIM	Y	[34, 37]
**Mukherjee_HOS/CDDP-P**	CDDP	2.2	–	5-FU	Y	[41]
**Partial MAP resistance**						
**Serra_U2OS/DOX-C**	DOX	330	–	MTX	Y	[34]
**Serra_SAOS2/DOX-C**	DOX	338	–	MTX	Y	[34]
**Shen_U2OS/DOX-P**	DOX	95.6	–	CDDP, PAC	Y	[44]
**Shen_MG63/DOX-P**	DOX	72.9	–	CDDP, PAC	Y	[44]
**Wang_SAOS2/MTX-P**	MTX	12.73	CDDP	DOX, IFOS, EPI, THP	N	[49]
**Complete MAP resistance**						
**Serra_SAOS2/MTX-C**	MTX	281	–	DOX, CDDP, TRIM	Y	[34, 37]
**Lourda_U2OS/DOX-C**	DOX	15	–	MTX, CDDP	Y	[72]
**Lourda_KHOS/DOX-C**	DOX	6.3	–	MTX, CDDP	Y	[72]
**Han_SOSP9607/CDDP-P**	CDDP	6.25	PAC	MTX, DOX	N	[54]
**Wang_MG63/DOX-C**	DOX	10.53	–	MTX, CDDP, IFOS	Y	[58]
**Xu_MG63/DOX-C**	DOX	6	–	MTX, CDDP	*NA*	[65]

Chemoresistant models were generated by exposure to a single MAP agent. Cross-resistance to further MAP drugs, in addition to non-MAP drugs such as (TRIM) Trimetrexate, (PAC) Paclitaxel, (THP) Theprubicin, (EPI) Epirubicin, (5-FU) 5-Fluorouracil, was also investigated in some studies. Models with partial MAP resistance were determined to be cross-resistant to one further MAP drug and include those that were not investigated for complete MAP resistance. Complete MAP resistance identifies models that were determined to be resistant to all MAP agents. Expression of *ABCB1*/PGP is indicated compared to the respective parental control. (–) No further drugs tested, *NA* Not tested.

### Summary of differential expression analysis

This review collected data on changes in expression at the gene, protein and ncRNA level in chemoresistant OS models. This was performed in order to identify potential trends in the expression of resistance markers across methodologies and models. Six studies were discovery driven using techniques to validate resistance markers identified by microarray [43, 44, 46, 50, 51] or gene amplification and copy number studies [34], whilst 30 studies were candidate driven. Expression is reported for; 80 genes in 30 models using qPCR [34, 36, 37, 41–44, 46–50, 52–61], 25 proteins in 19 models by western blot or flow cytometry [35, 36, 47, 53, 55, 57, 58, 61–66], and 25 ncRNAs in 16 models using qPCR [44, 51, 55, 59, 61, 62, 65–73]. Gene expression analysis was completed across all cell lines, strategies and MAP drugs whilst, protein and ncRNA analysis was not performed in the HOS, SOSP-9607 or 143b cell lines. DOX resistant models were always the most common, with 17 used for gene expression analysis, 14 for protein analysis and 11 for ncRNA analysis. One protein and two ncRNAs displayed a mixed expression profile dependent on the model and study.

### Comparison of in vitro resistance mechanisms and pathways

**Fig. 1:**
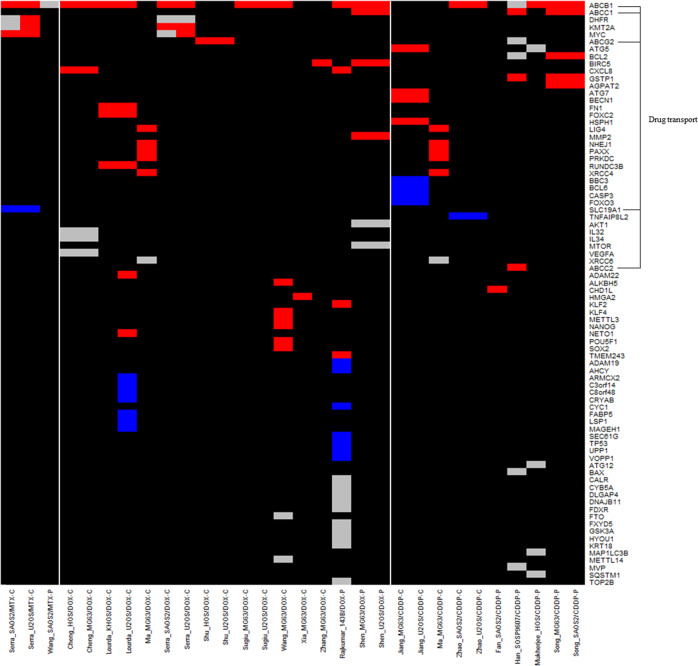
Differential gene expression in chemoresistant OS models. Gene expression data for in vitro studies was extracted into a data table and entered into R to develop a summary heatmap of gene expression for each distinct model. Genes are organised from most (top) to least (bottom) investigated across different models. Models are ordered by the MAP agent used for generation. (Blue) Downregulated, (Red) Upregulated, (Grey) Unchanged with respect to corresponding parental cell line, (Black) unknown or ambiguous results signifying an area of potential research interest. Genes involved in drug efflux are highlighted due to their occurrence (number of studies = 22).

**Fig. 2:**
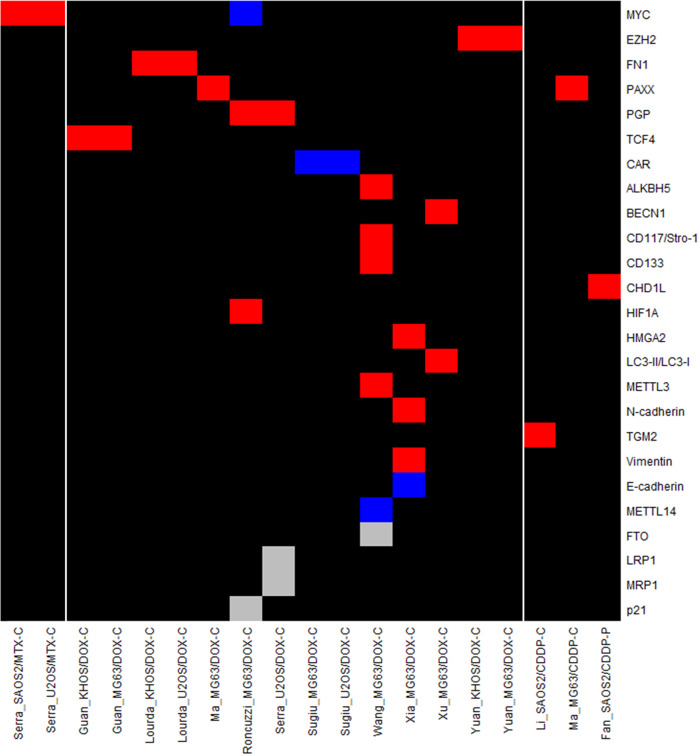
Differential protein expression in chemoresistant OS models. Protein expression data for in vitro studies was extracted into a data table and entered into R to develop a summary heatmap of expression grouped by distinct model. Proteins are organised from most (top) to least (bottom) investigated across different models. Models are ordered by the MAP agent used for generation. (Blue) Downregulated, (Red) Upregulated, (Grey) Unchanged with respect to each models’ corresponding parental cell line, (Black) unknown or ambiguous result signifying an area of potential research interest (number of studies = 13).

Altered drug transport was a popular resistance mechanism evaluated across studies and included both drug transporters such as *SLC19A1*/RFC1 and the ATP binding cassette family of efflux proteins such as *ABCB1*, *ABCG2*, *ABCC1* and *ABCC2* (Figs. 1 and 2). The most common resistance marker analysed in 14 studies across 23 models was *ABCB1*, also known as *MDR1*, which encodes p-glycoprotein (PGP) [34, 35, 41, 43, 44, 49, 50, 52, 54, 56–58, 60, 64]. Overexpression of *ABCB1*/PGP was induced by; both exposure strategies, those with an RI of 2.2–338, treatment with any MAP agent and in most cell lines explored. The RI exceeded clinical levels in 76% of models with *ABCB1* upregulation, specifically in 100% of DOX (RI = 6.3–338) and MTX (RI = 135–281) resistant models, whilst all CDDP-resistant models (RI = 2.2–5) were within the clinical range (RI ≤ 6.1). *ABCB1* was upregulated across all 14 DOX resistant models, 11 being continuously treated [34, 35, 50, 52, 57, 58] and three being pulsed [43, 44] and in 5/6 CDDP models [41, 54, 56, 60] and 2/3 MTX models [34, 49]. Downregulation of the intracellular transporter for MTX, *SLC19A1*/RFC1, was seen in two MTX resistant models [37].

There were several common resistance markers upregulated across models exposed to different MAP agents indicating a role in cross-resistance. In a CDDP resistant chondroblastic model with cross-resistance to MTX and DOX, overexpression of *ABCC1/*MRP1 and *ABCC2*/MRP2 but not *ABCG2* or *ABCB1/*PGP was observed [54]. ABCC1 has also been linked to DOX resistance alongside cross-resistance to CDDP and PAC [44], whilst *ABCG2* has been linked to DOX resistance in a further model although no cross-resistance testing was reported [48]. Out of the 21 models with *ABCB1*/PGP upregulation; 13 did not report cross-resistance, one showed no cross-resistance to DOX [34] and the remaining seven models were cross-resistant to 5-FU [41], DOX [34], MTX [34], PAC and [44] and MTX, CDDP and IFOS [58]. Drug efflux is therefore a common mechanism mediating cross-resistance in vitro and this has been evidenced in OS for *ABCB1/*PGP, *ABCC1/*MRP1 and *ABCC2/*MRP2. In both CDDP and DOX resistant MG63 models with clinically reported levels of resistance the expression of DNA ligase (*LIG4)*, protein kinase DNA activated catalytic subunit (*PRKDC)*, x-ray repair cross complementing 4 (*XRCC4), XRCC4* like factor (*NHEJ1/*XLF*)* and paralog of *XRCC4* and *XLF* (*PAXX)* was elevated, indicating the importance of DNA repair as a mechanism of shared DOX and CDDP resistance. Despite this, cross-resistance was not reported in either model to further validate this [47]. DOX and MTX resistant models shared upregulation of the global gene regulators *KMT2A/*MLL and *MYC* at both the gene and protein level [34, 36]; however, MYC was reduced at the protein level in one DOX resistant model [35].

**Fig. 3:**
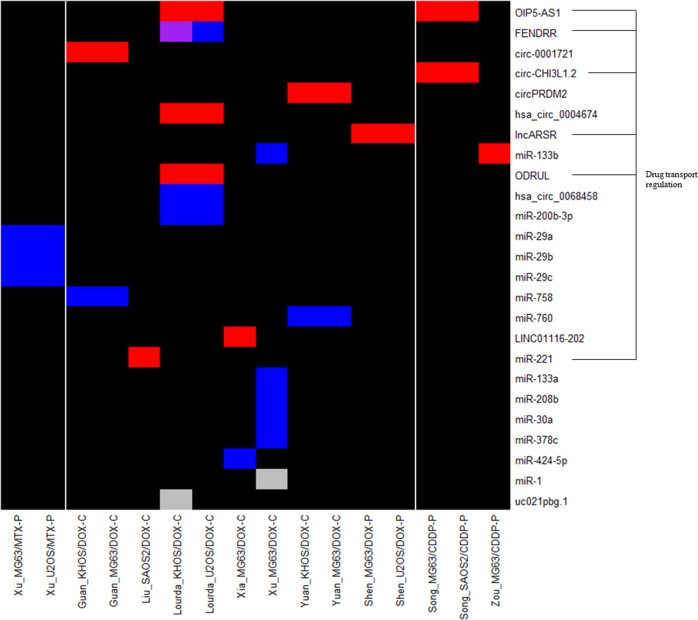
Differential ncRNA expression in chemoresistant OS models. ncRNA expression data for in vitro studies was extracted into a data table and entered into R to develop a summary heatmap of expression grouped by distinct model. ncRNAs are organised from most (top) to least (bottom) investigated across different models. Models are ordered by the MAP agent used for generation. (Blue) Downregulated, (Red) Upregulated, (Grey) Unchanged with respect to each models’ corresponding parental cell line, (Purple) Differing expression results within the same model, (Black) unknown or ambiguous result indicating an area of potential research interest. ncRNAs shown to be involved in the regulation of drug efflux proteins are highlighted due to their occurrence (number of studies = 15).

Several ncRNAs were identified to regulate the expression of common chemoresistance markers whilst others had mixed effects (Fig. 3). In one study *FENDRR* expression was unchanged [51] whilst in a further study it was downregulated and controlled the expression of *ABCB1/*PGP and *ABCC1/*MRP1 [71] despite these studies sharing the same model. Results also differed for *miR-133b* which was upregulated in CDDP resistance where it inhibited adduct formation [73] but downregulated in DOX resistance with an unknown mechanism [65], suggesting a dual role in resistance. The upregulation of long non-coding RNA *OIP5-AS1* induced resistance by sponging *miR-340-5p* allowing the expression of 1-acylglycerol-3-phosphate O-acyltransferase 2 (*AGPAT2*/LPAATβ). This in turn positively influenced the *PI3K/Akt/mTOR* axis and upregulated *ABCB1/*PGP*, ABCC1/*MRP1, glutathione-s-transferase 1 (*GSTP1*) and B cell lymphoma 2 (*BCL-2*) leading to CDDP resistance [56, 68]. Similarly, in two DOX resistant models with cross-resistance to CDDP and PAC, overexpression of the long non-coding RNA *lncARSR* regulated the expression of *ABCB1/*PGP, *ABCC1/*MRP1, matrix metalloproteinase (*MMP-2*) and survivin (*BIRC5*) via an *Akt* dependent mechanism [44]. Whilst in DOX resistant models upregulation of *OIP5-AS1* inhibited *miR-200b-3p* resulting in overexpression of fibronectin 1 (*FN1*) [61]. In DOX resistant models *circPRMD2* sponged *miR-760* leading to overexpression of the transcriptional represser enhancer of zeste homologue 2 (*EZH2*) [66] whilst *LINC01116* interacted with EZH2 to inhibit *miR-424-5p* causing upregulation of non-histone chromosomal mobility group AT hook (*HMGA2*) and the promotion of cell migration factors [55]. Autophagy was also regulated by ncRNAs with diminished levels of *miR-30a* in one model leading to the upregulation of autophagy markers beclin-1 (*BECN1*) and microtubule associated protein LC3-II [65].

### Expression analysis in chemoresistant variants

This review has highlighted that only a small number of studies investigated changes in the expression of resistance markers across variants with increasing levels of resistance. Expression of *MYC* was significantly upregulated in a highly DOX resistant U2OS model (RI = 330) but not in earlier variants (RI ≤ 58). In the same study, *KMT2A/*MLL overexpression was only observed in DOX resistant SAOS2 variants with high resistance (RI ≥ 105) [34]. The impact of RI on expression is further evidenced in an MTX resistant U2OS variant with a low clinically relevant level of resistance (RI = 3) that showed no change in *ABCB1/*PGP, *DHFR*, *KMT2A/*MLL or *MYC* expression [34]. However, as the RI increased to 21 upregulation of *DHFR* [34, 37] and *MYC* [34, 36] were observed and at an RI of 69 was followed by increased and decreased levels of *KMT2A/*MLL [34] and *SLC19A1/*RFC1 [37], respectively. Finally, at the highest RI of 135 expression of *ABCB1/*PGP became significant [34]. Similar results were also seen in an MTX resistant SAOS2 model where *MYC* expression became significant in variants with an RI ≥ 109 [34, 36] followed by *ABCB1/*PGP only in the most resistant variant (RI = 281) [34]. Differences were observed in this model compared to U2OS with *SLC19A1*/RFC1 significantly downregulated across all variants [37] whereas, there was no change in the expression of *DHFR* [34, 37] and *KMT2A/*MLL [34] across all variants. Conversely, a DOX resistant MG63 model showed overexpression of *ABCB1*/PGP and HIF1α and downregulation of MYC in both variants investigated (RI = 10–28) [35].

### Clinical chemoresistance studies

Relation of resistance factors identified in in vitro models to biomarkers linked to patient outcome is a necessary step for the validation and evaluation of current models in mimicking the patient experience. This data could be used to modify the methods used to generate in vitro models and increase the likelihood of incorporating clinically relevant chemoresistance mechanisms within drug screens. For example, factors identified in samples obtained at biopsy that predict a poor response to chemotherapy, relapse or metastasis may indicate mechanisms of innate resistance. In contrast, markers identified in surgical samples from poor responders that are absent in paired biopsy samples might indicate mechanisms of acquired resistance. These could then be used to determine if in vitro models mimic innate or acquired chemoresistance and lead to the identification of patient subsets most likely to benefit from the discovery of novel agents in these models.

### Patient demographic

The search strategy employed in this review identified 18 clinical studies published between 1995-2021 that assessed expression of one gene [74], 12 proteins [26, 28, 36, 48, 75–83] and ten ncRNAs [82, 84–87] in relation to relapse, metastasis and response to MAP therapy in OS patients (Table 4). Eight of these proteins overlapped with factors investigated in cell models of chemoresistance. Seven studies investigated more than one patient outcome measure, two studies used both biopsy and surgical samples and one study used biopsy and relapsed samples. Response was used as a patient outcome measure in 17 studies and all were defined by the standard percentage necrosis threshold [26, 28, 36, 48, 74–82, 84–87]. Relapse as a patient outcome measure was variably defined with 1/7 studies using local relapse only [81], 2/7 studies including relapse at any site [77, 79] and the remaining studies providing no clear definition [36, 76, 80, 83]. Metastasis was used as a patient outcome measure in two studies that clearly stated pulmonary metastasis [81, 87]. Six studies were conducted in Italy, seven in China, three in Japan and one in Israel and Iran. Reporting of patient age varied with some studies including children and adolescents only and others including adults with a maximum stated age of 81. Patients were more commonly male and five studies matched gender. The majority of studies included patients with diverse subtypes, high grade and stage A-B OS of the extremities. Where clearly reported, OS was typically newly diagnosed, operable and non-metastatic at diagnosis. Commonly patients were treated with neoadjuvant combined hdMAP/hdMAPi, although one study used adjuvant DOX treatment alone. The number of samples in each study ranged from 6 to 149.

**Table 4:** Patient characteristics and outcome summary of clinical studies.

Reference	Year	Study location	Age	Gender (F:M)	Sample characteristics	Patient characteristics	Treatment	Sample number	Sample type	Expression analysed	Method	Outcome measure	P<0.05
[75]	1995	Italy	47% ≤14, 53% 15-39	1.04: 1	Stage IIA-IIB, High grade	ND, NM, Op, EOS	hdMAP	92	PRE	↑PGP, *ATP-dependent efflux protein*	IHC	Necrosis	No
[83]	1999	Italy	54% ≤14, 46% >14	1: 1.18	Grade III-IV, Mixed subtype	ND, NM, Op, EOS	Post-op DOX	37	PRE	↑PGP, *ATP-dependent efflux protein*	IHC	Relapse	Yes
[84]	2020	China	53% <18, 47% ≥18	1: 1.4	Stage IIA-IIB, Mixed subtype	ND, NM, Op, EOS	hdMAP	72	PRE	↓Circ-LARP4, *gene regulation*, miR-424 sponge	qPCR	Necrosis	Yes
[76]	2003	Israel	15-67	Mixed	Matched	EOS	hdMAP	6	PRE vs R	↑MRP1, *ATP-dependent efflux protein*	WB	Relapse	No
										↑RFC, *MTX entry*			No
			11-81	1: 2.1	Mixed subtype			20		↑MRP1, *ATP-dependent efflux protein*			No
										↑RFC, *MTX entry*			Yes
				1: 2				9	PRE	↓RFC1, *MTX entry*		Necrosis	Yes
[74]	2021	Japan	<21	1: 2.83	High grade, Mixed subtype	EOS	MAPi	24	PRE	↑CDK4, *Cell cycle regulator*	qRT-PCR	Necrosis	Yes
[85]	2016	Japan	Mean 15, 10-25	1: 1	Osteoblastic	EOS	MAP	8	PRE	↑miR-100, *Post-transcriptional gene regulation*	qRT-PCR	Necrosis	Yes
										↑miR-125b, *Post-transcriptional gene regulation*			Yes
										↑miR-483-3p, *Post-transcriptional gene regulation*			No
										↑miR-124, *Post-transcriptional gene regulation*			No
										↑miR-127-3p, *Post-transcriptional gene regulation*			No
										↓miR-887, *Post-transcriptional gene regulation*			No
			Mean 14, 9-22	1: 1.22	Mixed subtype			20		↑miR-125b, *Post-transcriptional gene regulation*			Yes
										↑miR-100, *Post-transcriptional gene regulation*			Yes
[28]	2001	China	Mean 18, 4-16	–	Stage II-B	–	hdMAP	45	PRE	↑PGP, *ATP-dependent efflux protein*	IHC	Necrosis	No
									POST				Yes
[86]	2018	China	Matched	1: 1	Stage matched	–	MAP	42	POST	↓miR-377, *Post-transcriptional gene regulation*, Targets apoptosis regulator XIAP	RT-PCR	Necrosis	Yes
[77]	2009	Iran	20% ≤12, 80% 13-39	1: 2.33	Stage IIB, High grade, Mixed subtype	ND, NM, Op, EOS	hdMAPi	30	PRE	↑PGP, *ATP-dependent efflux protein*	IHC	Relapse	Yes
								15				Necrosis	No
[36]	2008	Italy	<40	–	High grade	ND, NM, EOS, COS	hdMAPi	61	PRE	↑DHFR, *Folate metabolism*	IHC	Relapse	No
													Yes
										↑c-MYC, *Transcription factor*			Yes
												Necrosis	No
										↑PGP, *ATP-dependent efflux protein*			No
													No
[78]	1995	Italy	–	–	Grade III-IV, Mixed subtype	NM, Op, EOS	hdMAPi	36	–	↑PGP, *ATP-dependent efflux protein*	SQ-IF	Necrosis	No
[79]	2003	Italy	24% ≤12, 76% 13-39	1: 1	High grade, Mixed subtype	ND, NM, Op, EOS	hdMAP	149	PRE	↑PGP, *ATP-dependent efflux protein*	IHC	Relapse	Yes
												Necrosis	No
[80]	2006	Italy	19% ≤12, 81% 13-39	1 : 1.56	High grade, Mixed subtype	ND, NM, Op, EOS, COS	hdMAPi	94	PRE	↑PGP, *ATP-dependent efflux protein*	IHC	Necrosis	No
												Relapse	Yes
[48]	2021	China	50% ≤20, 50% >20	1.13: 1	Grade I-III, Mixed subtype	–	MAP	68	POST	↑ABCG2, *ATP-dependent efflux protein*	IHC	Necrosis	Yes
[26]	1997	Japan	Mean 16.2, 5-30	1: 1.5	Stage IIA-III, Grade III-IV, Mixed subtype	EOS	hdMAPi	54	PRE	↑MT, *Zinc binding protein*	IHC	Necrosis	No
										↑GST, *Glutathione transferase*			No
										↑HSP27, *Chaperone protein*			No
										↑LRP, *Co-receptor for Wnt pathway*			No
								60	POST	↑MT, *Zinc binding protein*			Yes
										↑GST, *Glutathione transferase*			Yes
										↑HSP27, *Chaperone protein*			Yes
										↑LRP, *Co-receptor for Wnt pathway*			Yes
[81]	2017	China	66% ≤20, 44% >20	1: 1.71	Stage I-IV	Op, EOS	hdMAPi	133	–	↑PTN, *Growth factor*, Regulates PGP expression via ALK/GSK3β/β-catenin pathway	IHC	Local relapse	Yes
												Necrosis	Yes
												Lung metastasis	No
[82]	2014	China	Mean 9.6, 7-14, Matched	1: 1	Stage IIA-III, Matched	Op, EOS	hdMAP	12	POST	↓TWIST-1, *Transcription factor*	WB	Necrosis	Yes
			6-16	1: 1.7	Stage IIA-III			70		↑miR-33a, *Post-transcriptional gene regulation*, Downregulates TWIST1	qRT-PCR		Yes
										↓TWIST-1, *Transcription factor*	WB		Yes
[87]	2018	China	70% <25, 30% ≥ 25	1: 1.43	Stage I-III	Op	MAPi	80	–	↑circPVT1, *Gene regulation*, Regulator of PGP expression	qRT-PCR	Necrosis	Yes
												Lung metastasis	Yes

Data was extracted from clinical studies included in this review. Treatment is shown for neoadjuvant regimen unless otherwise stated. Doses of each drug and number of cycles may vary and were not recorded. Due to variability in reporting patient age is recorded as mean with range, % in each age group, matched or < or >. The common function of each resistance factor is indicated in italics with any proposed functions as listed in each study recorded after. (Pro) Prospective, (Ret) Retrospective, (ND) Newly diagnosed, (NM) Non-metastatic, (Op) Operable, (EOS) extremity OS, (COS) central OS, (PRE) Pre-treatment, (R) Relapsed, (POST) Post-treatment, (IHC) Immunohistochemistry, (SQ-IF) Semi-quantitative immunofluorescence, (WB) Western blot, (–) Not clearly stated.

### Response

Seventeen studies included in this review investigated differential expression of one gene, 11 proteins and ten ncRNAs in relation to patient response defined by necrosis [26, 28, 36, 48, 74–82, 84–87]. One study used a preliminary and validation cohort [82] whilst a second study used an initial osteoblastic cohort followed by a validation cohort with mixed subtypes [85]. The type of tissue sample varied with nine studies using biopsy samples, three using surgical samples, two using both and a further three not clearly stating their sample type. Across all studies sample size varied from 8-149 with all patients treated with MAP(I)/hdMAP(I).

Downregulation of *circ-LARP4* in biopsy samples was reported more frequently in those later classed as poor responders (*P* = 0.032) [84]. *CircPVT1* was upregulated in poor responders by analysis of 80 OS samples (*P* = 0.025) [87]. In eight osteoblastic biopsy samples *miR-125* and *miR-100* were upregulated by RTq-PCR in poor responders whilst no significant difference in the expression of *miR-483-3p*, *miR-124*, *miR-127-3p* and *miR-887* was identified. In a validation cohort of 20 independent biopsy samples of mixed subtype the association of *miR-125* (*P* = 0.001) and *miR-100* (*P* = 0.002) with response was validated [85]. In 42 patient samples matched for age, stage and gender, *miR-377* was downregulated in poor versus good responders (*P* < 0.05) [86]. Matching of age, gender and stage was also used in another study involving 12 patients that found diminished levels of the osteogenic lineage transcription factor twist related protein 1 (TWIST-1) in poor versus good responders. This was then confirmed using a validation cohort of 70 patients in addition to enhanced *miR-33a* expression (*P* < 0.001), with correlation between these factors suggesting the negative regulation of TWIST-1 by *miR-33a* [82].

There was no association found between the levels of MYC or DHFR and patient response [36]. The expression of the growth factor pleiotrophin (PTN) (*P* = 0.003) in 133 OS samples [81], cyclin dependent kinase 4 (CDK4) (*P* < 0.05) in 24 biopsy samples [74] and the efflux protein ABCG2 (*P* = 0.02) in 68 surgical samples [48] correlated with poor response. In one study reduced *SLC19A1*/RFC1 levels in biopsy samples predicted a poor therapy response however the inverse was observed in relapsed samples [76]. The importance of tissue sample type was also demonstrated with metal binding metallothionein protein (MT), *GSTP1*/GSTπ, heat shock protein 27 (HSP27) and *MVP*/LRP1 which were upregulated in surgical samples of poor responders but not at biopsy, suggesting involvement in acquired resistance [26]. Seven studies encompassing a total of 492 patients concluded that PGP status at biopsy did not correlate with MAP therapy response (Table 5) [28, 36, 77–80, 83]. In contrast, only one study using samples obtained at surgery found an association between *ABCB1*/PGP expression and chemotherapy response suggesting possible involvement in acquired resistance (*P* < 0.001) [28].

**Table 5:** Clinical analysis of PGP expression in relation to response and relapse.

Reference	Sample	PGP^+^ patients		Antibodies	Sampling method	PGP threshold	Statistical test	Outcome
		Relapse	No relapse					
[83]	PRE	75% (15/20)	12% (2/17)	JSB1, MRK16	2 samples per tumour, central and periphery	PGP^-^ = ≤10%, PGP^+^ = >10%	Log rank	*P* < 0.001
[77]	PRE	80% (12/15)	13% (2/15)	C219, JSB1	–	PGP^-^ = ≤10%, PGP^+^ = >10%	Two-tailed Fischer’s exact	*P* = 0.001
[36]	PRE	74% (17/23)	40% (15/38)	C494, JSB1, MRK16	–	–	Two-tailed Fischer’s exact	*P* = 0.01
[79]	PRE	55% (30/55)	18% (17/94)	C494, JSB1, MRK16	–	PGP^+^ = diffused immunostaining	Two-tailed Fischer’s exact	*P* < 0.0001
[80]	PRE	79% (27/34)	43% (26/60)	C494, JSB1, MRK16	–	PGP^-^ = ≤10%, PGP^+^ = >10%, using 2/3 antibodies	Two-tailed Fischer’s exact	*P* = 0.001
		**Poor responder**	**Good responder**					
[75]	PRE	24% (6/25)	33% (22/67)	C219, JSB1, MRK16	–	PGP^-^ = ≤10%, PGP^+^ = >10%	Log rank	*P* = 0.46
[28]	PRE	48% (12/25)	20% (4/20)	JSB1	–	PGP^-^ = ≤25%, PGP^+^ = >25%	Fischer’s exact	*P* = 0.066
[28]	POST	88% (22/25)	0% (0/20)	JSB1	–	PGP^-^ = ≤25%, PGP^+^ = >25%	Fischer’s exact	*P* < 0.001
[77]	PRE	55% (6/11)	75% (3/4)	C219, JSB1	–	PGP^-^ = ≤10%, PGP^+^ = >10%	Two-tailed Fischer’s exact	*P* = 0.604
[78]	PRE	32% (8/25)	45% (5/11)	C219, JSB1, MRK16	300 tumour cells	PGP^-^ = ≤15%, PGP^+^ = >15%	–	NP
[79]	PRE	29% (13/45)	33% (34/104)	C494, JSB1, MRK16	–	PGP^+^ = diffused immunostaining	Two-tailed Fischer’s exact	*P* = 0.62
[80]	PRE	55% (44/80)	64% (9/14)	C494, JSB1, MRK16	–	PGP^-^ = ≤10%, PGP^+^ = >10%	Two-tailed Fischer’s exact	NP

PGP upregulation in biopsy samples can predict patient relapse but not chemotherapy response defined by necrosis. The number and percentage of PGP^+^ patients within each patient outcome category is shown. *PRE* Pre-treatment, *POST* Post-treatment, (--) Not clearly stated, *NP* No *P* value stated.

### Relapse

Seven studies included in this review investigated differential expression of six proteins in relation to patient relapse [36, 76, 77, 79–81, 83]. The choice of tissue varied with five studies using biopsy samples, one comparing biopsy and recurrent samples in both matched and unmatched patients and one using pre-chemotherapy surgical samples. Similarly, sample size varied from 6 to 149 OS patients (mean = 72, *N* = 7). Of these studies 6/7 included patients treated with hdMAPI and one included patients treated only with post-operative DOX.

Overexpression of PTN (*P* = 0.001) in 133 samples [81], MYC (*P* = 0.001) in 61 biopsy samples [36] and *SLC19A1*/RFC1 (*P* = 0.00048) in 20 matched recurrent samples [76] increased chance of relapse. Levels of DHFR in biopsy samples [36] and *ABCC1*/MRP1 in matched samples [76] were not associated with relapse. Five studies investigated *ABCB1*/PGP expression with all concluding that upregulation of PGP at biopsy was linked to a greater chance of relapse [36, 77, 79, 80, 83].

### Metastasis

Two studies investigated the expression of a single protein and circRNA in relation to pulmonary metastasis [81, 87]. The tissue sample type was not clearly stated in either study. Overexpression of *circPVT1* in 80 samples was linked to greater chance of pulmonary metastasis (*P* = 0.038) [87] whilst no association was found for PTN expression in 133 samples (*P* = 0.109) [81].

### Review limitations

There was a lack of standard reporting on the methodology used for model development and future research should aim to clearly state this in order for its contribution to resistance to be evaluated. In rare cases researchers indicated that the chemoresistant models used in their study were previously generated, however provided no reference or name of a collaborator who may have provided these cell lines. This hinders the process of data accumulation and systematic analysis as these models are regarded as distinct when in fact data relating to this model should perhaps be collated with other studies that share the same model. For clinical studies the method of analysing percentage necrosis which has been shown to vary between pathologist and affect response classification [88] was not taken into consideration due to a lack of standard reporting. To overcome this, studies should in future adopt a standardised approach for deriving percentage necrosis such as the thorough method reported in Hauben et al. [89]. Similarly, determination of marker expression was not standardised with studies using varying thresholds for immunohistochemical analysis. In addition, due to its narrative and summative nature, this review solely focused on which factors were involved in chemoresistance regardless of their degree of influence and a meta-analysis could be pursued to determine the contribution of each of the markers discussed.

## Discussion

Over the past 40 years research into the use of chemoresistant models as tools for drug discovery has increased dramatically. In contrast, the treatments available to OS patients have not changed over the same period despite up to 68% of patients regarded as poor responders to first-line treatment [26] and no established second-line therapy available for these patients resulting in a poor prognosis [10]. Due to the rarity of OS within the population, reliable cell models are vital to identify novel treatments. There are currently many chemoresistant OS models generated via multiple strategies, however research must now focus on evaluating their clinical relevance in order to best utilise them as tools for drug discovery. As new versions of MTX thought to bypass key mechanisms of chemoresistance demonstrate a mere 13% response [14] and as the average cost of R&D for a single new anti-cancer drug was recently estimated as over $4460 million [90] this is a necessary step to evaluate the clinical relevance of existing pre-clinical models in order to improve their development and use.

From the literature surveyed all of the models were generated from established cell lines likely due to their ease of availability compared to primary OS cell lines. The three most commonly used cell lines (MG63, U2OS and SAOS2) were derived from paediatric Caucasian patients and were of epithelial or fibroblastic origin; however, this may not imitate the demographic of patients shown to have a higher incidence. For example, Black patients have been shown to have a higher incidence across all age groups [3], therefore the inclusion of the only ATCC available OS cell line derived from a Black patient (SJSA-1, 19 years, male) could be considered to ensure more accurate patient representation. Despite the use of established cell lines derived from patients with a known histological subtype, today many of these cell lines remain disputed. For example, based on gene signature analysis MG63 cells originating from a fibroblastic OS patient were predicted as osteoblastic [91], whilst a further study found they could only differentiate into cells of the chondrogenic lineage [92]. Differences in the characteristics and morphology of these cell lines may be attributed to batch variation, however relation to histological subtype may be an important consideration as studies have shown these respond differently to therapy. In a study involving 272 patients treated with MAP, the telangiectatic and fibroblastic subtypes had the highest 5-year survival and complete response rate, whilst chondroblastic scored the lowest [93]. In contrast, a study in 3482 patients demonstrated similar 5-year survival rates amongst all subtypes in patients under 24 years old, although differences were observed in patients aged over 24 years indicating age may play a role in this disparity [3]. This highlights a potential link between subtype and patient outcome; however, despite chondroblastic being the second most common subtype only one study found via this review used a cell line originating from a chondroblastic patient [54]. More diverse cell lines should be included that mimic the frequency of patient subtypes to determine if molecular mechanisms of chemoresistance change based on subtype or whether differences in patient outcome are linked to variation in drug perfusion through the changing matrix compositions.

In order to establish in vitro models of chemoresistance that resemble patient chemoresistance, the clinically relevant mechanisms of action of a drug needs to be induced. For example, 2 µM is regarded as the maximum initial peak plasma concentration for DOX with this decreasing within 1 h to 25–250 nM and it has been suggested that greater concentrations may induce mechanisms of drug action that are not achieved clinically [21]. In three DOX resistant models the final concentration exceeded this by up to 50-fold [43, 47, 67], indicating alternative mechanisms of action may have been induced leading to potentially less clinically relevant resistance mechanisms. The level of resistance across all models and variants was much greater (RI 2.2–338) [34, 41] than found in chemoresistant cancer patients (RI 2–5) [30] and specifically in sarcoma patients (RI ≤ 6.1) [31]. As the RI may influence multi-drug resistance and the expression of intrinsic resistance markers, as seen by the use of variants in studies included in this review, this indicates that the majority of models may stimulate resistance mechanisms that are not clinically relevant. From reviewing the literature there appears to be a lack of consideration for a development strategy that generates a clinically relevant OS model by use of peak plasma drug concentrations, pulsed exposure and the development of a low resistance level.

By far the most studied marker across both in vitro and clinical studies was *ABCB1*/PGP, which was greatly upregulated in models generated by any MAP drug. This contrasts with a clinical report that indicates PGP is a mechanism associated only with DOX resistance, with PGP^+^ patients benefitting from treatment with MTX and CDDP [83]. In a single small study PGP was also not involved in the DOX resistance of sarcoma patients with a resistance level of ≤6.1 with evidence instead pointing towards a detoxification system such as GST that scavenges free radicals [31]. In variants identified by this review there was a trend for greater PGP expression as cells developed greater levels of resistance. This is in line with prior studies that imply PGP expression develops as a later stage of model development, suggesting this may be a mechanism of acquired resistance linked only to highly resistant cells. This finding was initially demonstrated in DOX resistant murine erythroleukemia cells where the C7D and PC4 cell lines exhibited PGP upregulation only when an RI of 12 and 98, respectively, was achieved. It was also shown that treatment with a low dose for prolonged periods failed to induce PGP overexpression, suggesting there may be a specific concentration or resistance threshold that must be met and that this may be cell line specific. In the same study, cross-resistance to vincristine and anthracycline via reduced uptake occurred prior to PGP overexpression suggesting that PGP is not critical for a multi-drug resistant phenotype [94]. This mirrors two studies in this review with *ABCB1* overexpression in MTX [49] and CDDP [54] pulsed models not required for cross-resistance to DOX, MTX or IFOS.

With 40% of OS patients expressing moderate to high levels of PGP at diagnosis it has the potential to be an important mechanism of innate resistance [95]. However, evidence for the involvement of PGP in response, metastasis, recurrence and survival is conflicting. In this review PGP upregulation at biopsy was not associated with a poor response to MAP therapy but was able to predict relapse. One possible explanation for the association between PGP and relapse but not chemotherapy response provided by Serra et al. [79] suggests that PGP status and necrosis identify two distinct patient subsets, with percentage necrosis an indicator of resistance to combined treatment with PGP and non-PGP substrates such as MTX. PGP expression on the other hand may represent a subset of resistance to PGP substrates such as DOX in addition to disease progression. In a meta-analysis encompassing 631 OS patients across 14 clinical studies treated with various regimens, PGP expression was only associated with disease progression defined as relapse, metastasis or death and not histological response to chemotherapy and this mirrors the findings of this review [96]. In three studies included in this review there was a link between PGP expression and disease progression. The first study indicated an elevation of PGP levels at biopsy in grade 4 but not grade 3 tumours [75] and a second showed a relationship between PGP status at biopsy and advanced grade [78]. Finally, a third study linked PGP expression at biopsy to high mitotic activity and adverse events defined by relapse at any site or death during remission [77]. Together this provides a basis for the use of elevated PGP levels at biopsy as a marker for disease progression rather than MAP response.

There is mixed evidence in the literature for the relationship between *ABCB1*/PGP and aggressiveness in vitro. In the widely used DOX resistant variants developed by Serra et al. (1993) [97] both *ABCB1* expression and the population doubling time increased with increasing resistance, with pulmonary metastasis diminished in a xenograft model. Similarly, in DOX cross-resistant variants generated by vincristine exposure, the level of *ABCB1* increased with increasing resistance whilst the growth rate and migratory potential diminished [98]. This reduced aggressiveness could be attributed to additional mutational burden however, transfection of *ABCB1* in the U2OS cell line eliminated tumour formation and lung metastases in a subcutaneous xenograft model and was causally linked to reduced aggressiveness. In this study, clones with differing levels of PGP expression showed increased resistance to DOX (RI = 10–50), actinomycin D and vincristine but not MTX or CDDP as PGP levels increased [99]. This demonstrates that *ABCB1* induced by transfection or DOX exposure is associated with high levels of resistance to PGP substrates and a less aggressive phenotype in vitro and in vivo and this directly contrasts with clinical data suggesting PGP as an indicator of aggressiveness. However, in one study a variant induced by vincristine exposure with a low RI (DOX/MTX = 2.48) and relatively low *ABCB1* expression compared to later variants showed a slight increase in growth and a significant increase in migratory potential compared to the parental cell line [98]. A model with a clinically relevant level of resistance mediated in part by *ABCB1* therefore induced a more aggressive phenotype in vitro and this is in line with the association between PGP and high grade in OS patients. Further evidence for this can be seen in clinical OS data via the R2 Genomics platform whereby *ABCB1* expression is linked to poorer metastasis-free survival (*P* = 0.046, *n* = 88) but not overall survival (*P* = 0.068, *n* = 88). In summary, there may be a link between *ABCB1* expression, high resistance to PGP substrates and reduced aggressiveness; however, clinically *ABCB1* is not a key indicator of chemoresistance to combined therapy yet is associated with disease progression that has so far only been reflected in a model with low resistance. A reliance on models with excessive RIs may therefore be confusing the field of chemoresistance by altering the phenotype and overexaggerating the impact of genes like *ABCB1* in clinical chemoresistance. Overall, the data regarding a direct link between *ABCB1* expression and aggressiveness in in vitro models is conflicting and this warrants further investigation in variants with increasing resistance.

The above observations may explain why the use of PGP inhibitors (PGPi) has so far been unsuccessful in the clinic. First generation PGPi verapamil failed to elicit a response and induced cardiotoxicity in chemoresistant ovarian cancer patients [100]. Second generation PGPi, with improved potency and minimised side effects, were abandoned due to cross-over between cytochrome p450 and PGP substrates that altered drug pharmacokinetics. Third generation inhibitors showed increased potency and reduced cytochrome p450 interactions, one example being tariquidar which inhibits PGP via non-competitive binding [101]. However, a partial response was only seen in 8% (4/48) of cancer patients after tariquidar and docetaxel treatment, with retention of the radiotracer and PGP substrate ^99m^Tc-sestamibi varying between patients. One possible explanation for the poor response observed is that PGP status was not assessed in this patient group, suggesting other mechanisms of drug resistance dominated and were unaffected by PGP inhibition [102]. Similar response rates were also seen in multi-drug resistant breast cancer, with a mere 6% (1/17) partial response rate after treatment with tariquidar and chemotherapy. In this study, only 36% (5/14) of patients were PGP^+^ therefore it was not a key mechanism of multi-drug resistance and even amongst PGP^+^ patients only 20% showed a modest benefit from inhibition [103]. In addition, two phase III trials (NCT00042315/NCT00042302) using tariquidar in combination with chemotherapy in non-small cell lung cancer were terminated due to bone marrow toxicity [102], indicating that even modern PGPi exert significant adverse effects. An explanation for this lies in a potential role for PGP in healthy bone with 22% of normal bone marrow samples being PGP^+^ [104] and expression also seen within the growth plate of healthy bone [105]. Furthermore, in erythroleukemia cells with PGP overexpression, inhibition by verapamil reduced DOX resistance towards levels seen in earlier PGP^-^ variants indicating that early resistance mechanisms persist in later variants and are not directly associated with PGP [94]. Together this highlights the conflicting evidence surrounding PGP in OS patient response, its key role in chemoresistance in current in vitro models and its apparent undruggability. Overall, this suggests that there may be a disparity between chemoresistant models and clinical data in regards to PGP. There is a possibility that PGP may be involved as a late mechanism of acquired resistance explaining its link with disease progression in patients rather than response. Future strategies of model development may therefore seek a lower RI in order to detect earlier clinically relevant resistance mechanisms.

This review also found limited evidence in the literature pertaining to possible resistance mechanisms for CDDP and MTX. In one study there was no change in the expression of *ABCB1*/PGP or *MVP*/LRP1 in a pulsed CDDP resistant chondroblastic model with cross-resistance to MTX and DOX [54]. With levels of *MVP*/LRP1 in surgical but not biopsy samples associated with poor response in patients this model may mimic early and clinically relevant mechanisms of resistance through the absence of both *MVP*/LRP1 and *ABCB1*/PGP expression [26]. The overexpression of *GSTP1* in this model and two further pulsed CDDP resistant models with low resistance [54, 56] suggests a role for drug detoxification in OS chemoresistance. Evidence for this role in patients was seen in one study in surgical but not biopsy OS samples from poor responders [26]. Additive and synergistic results have been observed in OS with the GST inhibiter NBDHEX in combination with CDDP and DOX, but not MTX, implying a potential overlap in resistance mechanisms induced by DOX and CDDP [106]. There is also in vitro evidence to suggest cross-resistance to CDDP and DOX is linked to enhanced DNA repair [47], autophagy [46, 65] and drug efflux via transporters such as *ABCC1/*MRP1 [44, 54]. However, *ABCC1*/MRP1 levels did not change between samples obtained at biopsy and recurrence suggesting no involvement in acquired multi-drug resistance in patients [76]. Further work is needed to clarify the supposed involvement of autophagy and DNA repair markers in MAP resistance in clinical samples.

Innate resistance was explained by reduced accumulation of MTX through the downregulation of the MTX transporter *SLC19A1/*RFC1 in biopsy samples, whilst overexpression was recorded in relapsed samples. An explanation for this disparity by the authors suggests the extreme doses administered and high concentrations (1 mM) reported in the blood after infusion may allow MTX to enter cells through passive diffusion. As an adaptive mechanism to avoid total DHFR inhibition cells upregulate *SLC19A1*/RFC1 to promote the accumulation of the folate rescue drug leucovorin administered alongside MTX to allow cell division to continue [76]. A study commonly quoted within the field suggests that 65% of OS samples show downregulation of RFC1 at biopsy with this proportion reducing to 45% in surgical and relapsed samples implying a potential involvement of decreased MTX entry in innate resistance [107]. In MTX resistant models there was a trend for decreasing *SLC19A1*/RFC1 expression with increasing RI linking decreased MTX accumulation with greater resistance [37]. As these models mimic the results found in a small sample of biopsy samples they replicate a model of innate resistance. Nevertheless, reduced *SLC19A1*/RFC1 levels in patients may not fully explain MTX resistance as TRIM, which enters cells through an alternative mechanism, has a low response rate in relapsed OS patients [14]. This may also explain how upregulation of DHFR, the target of both MTX and TRIM, failed to predict MAP response or relapse in OS biopsy samples [36]. In this review, overexpression of *DHFR* was observed in a highly resistant U2OS model with cross-resistance to DOX; however, no change was seen in an SAOS2 model with cross-resistance to both DOX and CDDP [34, 37]. Similarly, in DOX resistant U2OS and SAOS2 models *DHFR* was not upregulated despite cross-resistance to MTX [34]. Target upregulation is therefore a mechanism of acquired MTX resistance in a single highly resistant OS model yet can develop by alternative methods that are shared in DOX and MTX multi-drug resistance. Expression of the transcription factor *MYC* increased with increasing resistance in MTX resistant models [34, 36], was upregulated in one DOX resistant model with excessive resistance (RI = 330) [34] yet was downregulated in another DOX resistant model [35]. As patients with high levels of MYC at biopsy were more likely to relapse yet no difference was observed in MAP response [36] these models may better reflect disease progression. In the scope of this review only five MTX resistant models were investigated despite previous reports establishing paediatric patients as MTX resistant yet responsive to CDDP and DOX. These patient derived cells also had similar levels of resistance in samples obtained pre- and post-chemotherapy [108]. Together this proposes a need for more models of MTX resistance in OS specifically to investigate innate resistance.

Identifying ncRNAs involved in chemoresistance appears promising due to the ability to identify whole regulatory pathways involved in resistance. In one in vitro study *OIP5-AS1* sponged *miR-340-5p* promoting the LPAATβ/PI3K/Akt/mTOR pathway which regulated the expression of *ABCB1* and *ABCC1* resulting in CDDP resistance [68]. A similar role was found for circ-*CHI3L1.2* in CDDP resistance with knockdown decreasing the levels of *ABCB1*, *ABCC1*, *GSTP1*, N-cadherin and vimentin also via the regulation of *miR-340-5p*/LPAATβ [70]. Moreover, *lncARSR* was shown to promote DOX and CDDP multi-drug resistance through the regulation of *ABCB1*, *ABCC1*, *MMP2* and *BIRC5* expression via an Akt dependent pathway [44]. Conversely, the lncRNA *FENDRR* negatively regulated the level of *ABCB1* and *ABCC1*, with *FENDRR* downregulation seen in DOX resistant models [71]. This provides a basis for the promotion of common resistance mechanisms such as drug efflux through the ncRNA regulated modulation of the LPAATβ/PI3K/Akt/mTOR axis. Although no statement can be made about the expression of the same ncRNA amongst clinical and in vitro OS studies, further evidence can be found for their involvement in chemoresistance amongst other cancers. In the wider literature, *OIP5-AS1* has also been linked to CDDP resistance in OS through sponging *miRNA-377-3p* and upregulation of fos-related antigen 2 (*FOSL2)* involved in bone development [109] and in a clinical study from this review *miRNA-377* was downregulated in surgical samples from poor responders [86]. Similarly, in a clinical study identified by this review the proposed *ABCB1*/PGP regulator *circPVT1* was associated with a poor response to MAPi therapy and greater chance of metastasis in 80 patient samples [87]. A role for *circPVT1* in metastasis was also found in PAC resistant gastric cancer models where via sponging *miR-124-3p* it increased expression of the transcription factor zinc finger E-box binding homeobox (*ZEB1*), which negatively regulates E-cadherin thus promoting cell migration. C*ircPVT1* also modulated levels of *ABCB1*/PGP and GSTπ further supporting its role in chemoresistance and metastasis observed in OS [110]. Together this suggests that ncRNAs may play a key regulatory role in chemoresistance, with prediction of binding partners having the potential to identify multiple resistance markers and this area warrants further investigation in both OS variants and patient samples.

## Conclusion

OS is a challenging disease and despite life-altering surgery and intensive combined chemotherapy many patients are poor responders due to innate and acquired MAP resistance. This review highlighted that current chemoresistant OS models may erroneously identify mechanisms that are not clinically relevant. Relevant MAP resistant models are vital to prevent the waste of resources, funding and time and in order to benefit patients who urgently need new effective treatment options which have not changed in over 40 years. Multiple factors such as the exposure strategy, chemotherapy, concentration, cell line and RI may alter the resistance mechanisms developed. Future research should incorporate; a panel of cell lines representative of OS subtypes, variants with increasing RIs, well-defined treatment strategies, MAP cross-resistance testing and combined MAP exposure for comparison of resistance marker expression. These markers can then be compared against data from OS patients treated with MAP therapy in relation to response and the most relevant can be used in drug screening and development. Confirmation that markers identified at surgery reflect acquired resistance by comparison with paired biopsy samples is required to determine that the effect seen is treatment induced and not an innate resistance mechanism observed at baseline. This is critical to elucidate patient subsets with innate or acquired resistance that will benefit the most from addition of these novel agents to their treatment regime.

## Materials and methods

### Search strategy

This in-depth systematic review follows the Preferred Reporting Items for Systematic Reviews and Meta-Analyses guidelines [111]. The databases included were Ovid, PubMed, Scopus and Web of Science and these were scanned using the same search terms in the title and abstract using tools specific to each literature database. The search terms used were:

(osteosarcoma OR “osteogenic sarcoma” OR “bone sarcoma” OR “bone neoplasm” OR “primary bone cancer” OR “bone cancer”) AND (chemoresistant OR chemoresistance OR resistant OR resistance) AND (patient OR sample OR biopsy OR “cell line” OR “in vitro”) AND (methotrexate OR MTX or amethopterin OR otrexup OR rasuvo OR rheumatrex OR trexall OR mexate OR folex OR hdmtx) OR (cisplatin OR DDP OR CDDP OR platinol OR neoplatin OR “cis-diamminedichloridoplatinum(II)” OR cisplatinum OR cis-ddp OR platidiam OR platin) OR (DOX OR doxorubicin OR ADR OR adriamycin OR “14-hydroxydaunomycin” OR hydroxydaunorubicin OR rubex OR caelyx OR myocet OR doxil OR adriblastin).

### Eligibility

**Fig. 4:**
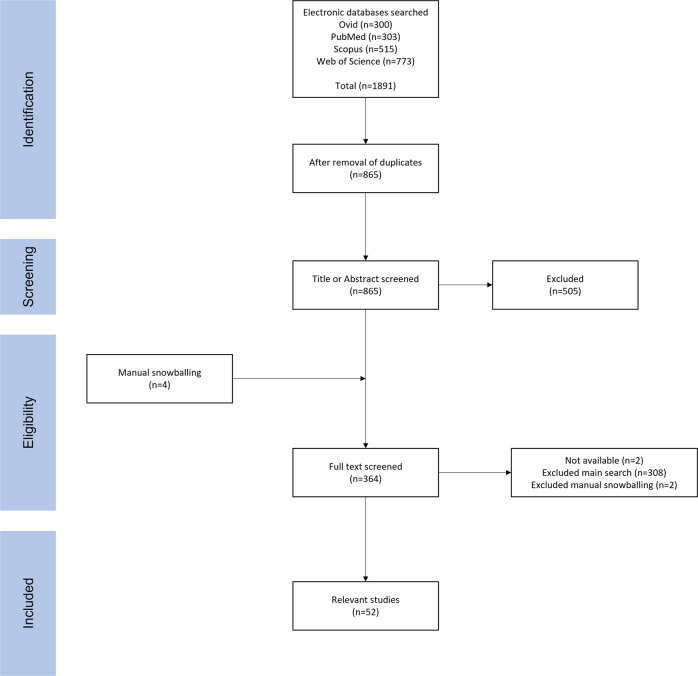
PRISMA screening flowchart. The search identified 1891 papers across four literature databases. The title and/or abstract of 865 articles were screened using the inclusion and exclusion criteria. The full text was assessed for the remaining 360 papers plus an additional four found via manual snowballing. A total of 310 articles were then excluded based on pre-determined criteria with a further two studies unavailable. A total of 52 papers were then included in this review.

The inclusion and exclusion criteria were pre-determined. This review focused on studies using OS patients or human OS cell lines published in English between 1970 and 2021. Conference abstracts, pre-prints and reviews were excluded. A thorough description of the screening process can be found Fig. 4 and the inclusion and exclusion criteria can be found in Supplementary 1. A specific DOX resistant MG63 model which has been widely used in this field was excluded from this review as a mutagen was used early in development that may induce molecular changes beyond that induced by MAP agents [112]. Reports where models were exposed only with MAP agents and resistant by determination of the EC_50_ or the RI were included. The RI is calculated using *RI* = *EC*50(*resistant*) ÷ *EC*50(*parental*) and therefore describes the fold-change in resistance.

For clinical studies this review aimed to identify resistance factors in OS patients treated with MAP/MAPi. Studies that investigated expression in both pre-treatment biopsy samples, post-treatment surgical samples or both are included and their sample type recorded. For clinical outcome measures, studies using the 90% necrosis threshold to signify poor and good responders were included along with those that used the analogous Huvos grading system (I + II = poor responders, III + IV = good responders). Further accepted patient outcome measures included relapse or metastasis.

### Risk of bias and quality assessment

The Cochrane Handbook was used to modify the pre-existing tools Toxicological Data Reliability Assessment Tool and the Office of Health Assessment and Translation risk of bias tool to generate a criteria for risk of bias and quality assessment [113–115]. The risk of bias criteria were further adapted from the risk of bias tools SYRCLE and ARRIVE, whilst the quality assessment criteria was also based upon the Newcastle-Ottawa scale and the National Institute of Health’s quality assessment tools (Supplementary 2 and 3) [116–119]. For risk of bias, studies were scored low, moderate or high and for quality assessment studies were assessed using a scoring system of 0–3 low, 4–7 moderate and 8–10 high, with studies graded as low excluded from the review. Both bias and quality were assessed independently by VLT and LT, discussed and final scores agreed upon.

### Data extraction

For in vitro studies information regarding: cell line, choice and concentration of inducing chemotherapy, diluent, EC_50_, strategy for model development, RI and cross-resistance were extracted into a data table made in Microsoft Excel. Genes, proteins and ncRNAs investigated for their differential expression between resistant and corresponding parental cell lines were recorded. For clinical papers the number of samples, treatment regimen and patient characteristics were noted, in addition to genes, proteins and ncRNAs analysed in relation to response, relapse and metastasis. For comparison of models, data regarded as non-significant by statistical analysis was recorded separately to those that reached significance. Only results that showed clear statistical analysis for data relevant to the inclusion criteria were included, with any ambiguous or unanalysed data excluded.

### Data analysis

The in vitro data table generated as outlined above was converted into.csv format and imported into RStudio using R version 4.1.0 (2021-05-18) by The R Foundation for Statistical Computing©. Figures were generated using the ggplot and heatmap functions. The use of the term ‘model’ was used to describe distinct chemoresistant in vitro models that were developed by a single group. This term was utilised to allow the creation of a profile of the differential gene, protein and ncRNA expression for each distinct model by summarising the data across studies that share these models. Models are named using the first author of the study they were originally generated from. Where variants were produced throughout model development with differing RIs, data is shown for the end model with the greatest RI with any variants discussed in text.

## Supplementary information

Inclusion exclusion criteria

Risk of bias

Quality assessment

## Data Availability

The publicly available R2: Genomics Analysis and Visualization Platform version 3.2.0 (http://r2.amc.nl) was accessed. The ‘Mixed OS (Mesenchymal)-Kuijjer-127-vst-ilmnhwg6v2’ dataset containing 88 osteosarcoma patient samples was used in relation to the gene *ABCB1* (ILMN_1812070) for metastasis-free and overall survival.
